# Liver Cancer Disparities in New York City: A Neighborhood View of Risk and Harm Reduction Factors

**DOI:** 10.3389/fonc.2018.00220

**Published:** 2018-06-14

**Authors:** Geetanjali R. Kamath, Emanuela Taioli, Natalia N. Egorova, Josep M. Llovet, Ponni V. Perumalswami, Jeffrey J. Weiss, Myron Schwartz, Stanley Ewala, Nina A. Bickell

**Affiliations:** ^1^Institute for Translational Epidemiology, New York, NY, United States; ^2^Tisch Cancer Institute, New York, NY, United States; ^3^Department of Population Health Science and Policy, Icahn School of Medicine at Mount Sinai, New York, NY, United States; ^4^Mount Sinai Liver Cancer Program, Divisions of Liver Diseases, Icahn School of Medicine at Mount Sinai, Tisch Cancer Institute, New York, NY, United States; ^5^Liver Cancer Translational Research Laboratory, BCLC, Liver Unit, Centro de Investigación Biomédica en Red de Enfermedades Hepáticas y Digestivas (CIBEREHD), Institut d’Investigacions Biomèdiques August Pi iSunyer (IDIBAPS), University of Barcelona, Barcelona, Catalonia, Spain; ^6^Institució Catalana de Recerca i Estudis Avançats, Barcelona, Catalonia, Spain; ^7^Division of Liver Diseases, Department of Medicine, Icahn School of Medicine at Mount Sinai, New York, NY, United States; ^8^Division of General Internal Medicine, Department of Medicine, Icahn School of Medicine at Mount Sinai, New York, NY, United States; ^9^Department of Surgery, Icahn School of Medicine at Mount Sinai, New York, NY, United States; ^10^Icahn School of Medicine at Mount Sinai, New York, NY, United States

**Keywords:** hepatocellular carcinoma, chronic hepatitis, health-care disparities, low-income populations, vaccinations, cancer screening

## Abstract

**Introduction:**

Liver cancer is the fastest increasing cancer in the United States and is one of the leading causes of cancer-related death in New York City (NYC), with wide disparities among neighborhoods. The purpose of this cross-sectional study was to describe liver cancer incidence by neighborhood and examine its association with risk factors. This information can inform preventive and treatment interventions.

**Materials and methods:**

Publicly available data were collected on adult NYC residents (*n* = 6,407,022). Age-adjusted data on liver and intrahepatic bile duct cancer came from the New York State Cancer Registry (1) (2007–2011 average annual incidence); and the NYC Vital Statistics Bureau (2015, mortality). Data on liver cancer risk factors (2012–2015) were sourced from the New York City Department of Health and Mental Hygiene: (1) Community Health Survey, (2) A1C registry, and (3) NYC Health Department Hepatitis surveillance data. They included prevalence of obesity, diabetes, diabetic control, alcohol-related hospitalizations or emergency department visits, hepatitis B and C rates, hepatitis B vaccine coverage, and injecting drug use.

**Results:**

Liver cancer incidence in NYC was strongly associated with neighborhood poverty after adjusting for race/ethnicity (β = 0.0217, *p* = 0.013); and with infection risk scores (β = 0.0389, 95% CI = 0.0088–0.069, *p* = 0.011), particularly in the poorest neighborhoods (β = 0.1207, 95% CI = 0.0147–0.2267, *p* = 0.026). Some neighborhoods with high hepatitis rates do not have a proportionate number of hepatitis prevention services.

**Conclusion:**

High liver cancer incidence is strongly associated with infection risk factors in NYC. There are gaps in hepatitis prevention services like syringe exchange and vaccination that should be addressed. The role of alcohol and metabolic risk factors on liver cancer in NYC warrants further study.

## Introduction

Cancer of the liver and intrahepatic bile ducts (liver cancer) is a public health problem in the United States (US). Since 1980, its nationwide incidence rate has tripled, and its mortality rate has doubled, outpacing the increase in any other cancer (2). Only 31% of those with localized liver cancer survive 5 years past diagnosis. The 5-year survival rate for regional and distant liver cancer is even poorer at 11 and 3%, respectively (2). Several studies have succeeded in reducing liver cancer incidence, most effectively through hepatitis B vaccination, and to an extent through antiviral therapy for hepatitis B and C (3). Although liver cancer prognosis can be improved by early detection and treatment during its long subclinical course, this is a challenge since liver cancer is usually asymptomatic in its early stages (3, 4). Currently, no guidelines currently exist for routine liver cancer screening in people of average risk; however, people at higher risk due to cirrhosis and/or chronic hepatitis B infection may benefit from screening with ultrasound exams, with or without alpha-fetoprotein blood tests, twice a year (5).

In New York City (NYC), liver cancer is the fifth leading cause of cancer-related death among men and seventh among women (1). The 2010–2014 age-adjusted liver cancer incidence rate in NYC was 12 per 100,000 residents, higher than the US (7.8) and New York State (NYS) (8.6) (1, 6). The age-adjusted mortality rate per 100,000 was also higher at 7.7 compared with the US (6.3) and NYS (6.1) (1, 6). Certain neighborhoods have incidence rates of 16–22.2 per 100,000, comparable to Asia, West Africa, and Central/South America (7).

New York City is a microcosm of the global population due to its unique demographics, high percentage of foreign-born inhabitants, and diversity of country of origin. Although a recent review examined racial/ethnic liver cancer disparities in the US (8), it has not been studied on a local level. A study of cancer incidence in NYC and three of its neighborhoods (East Harlem, Central Harlem, and Upper East Side) found that neighborhood was associated with incidence of all cancers, including liver cancer (9). To understand the basis for NYC disparities in liver cancer incidence and mortality, it is crucial to identify high-risk subpopulations, the risk factors most strongly associated with liver cancer, and how they are distributed in the city. The information can help inform preventive and treatment interventions for communities that require them the most.

## Materials and Methods

### Data Collection

Data were collected from the pool of adult (≥18 years) NYC residents (*n* = 6,407,022 per the 2010 US Census) at the neighborhood level, and defined neighborhood borders using NYC United Hospital Fund (UHF) codes. Originally, the UHF divided NYC into 42 distinct neighborhoods by combining adjoining zip codes areas with similar characteristics, meant to approximate NYC Community Planning Districts. To increase statistical power, these were later collapsed into 34 neighborhoods (10).

#### Primary Outcomes

Cancer data for NYC included age-adjusted incidence and mortality rates per 100,000 residents from the NYS Cancer Registry (1). The average incidence rate was calculated from the number of residents diagnosed with liver and/or intrahepatic bile duct cancer over 2007–2011, divided by the corresponding age-specific intercensal population estimates (from the NYS Department of Health). Age adjustment was based on the US Census 2000 standard population. Mortality rate estimates for 2015 were obtained from the NYC Department of Health and Mental Hygiene (NYCDOHMH) using the online interactive tool, Epiquery (11). Crude mortality rates are presented for neighborhoods with small numbers and/or unreliable age-adjusted estimates. For all outcomes, the most recent available estimates at the neighborhood level are presented.

#### Sociodemographics

Data on gender and race/ethnicity were collected from the 2010 US Census, and neighborhood-specific distributions were extracted using Epiquery. Data on poverty were obtained from the American Community Survey conducted by the US Census Bureau (12). Poverty was defined as the % of people reporting annual incomes below the federal poverty threshold during 2010–2014 ($11,139–$12,071 for one person). Data on insurance coverage were obtained from the Community Health Survey (CHS), an annual telephone survey conducted among NYC residents ≥18 years by the NYCDOHMH (13). We report the % of people who had no type of health insurance coverage.

#### Risk Factors for Liver Cancer

Viral hepatitis data are derived from surveillance reports filed by the Bureau of Communicable Disease (14). They include confirmed or probable cases of chronic hepatitis B and C reported to the Health Department by health-care providers and laboratories meeting the definitions by the Centers for Disease Control/Council of State and Territorial Epidemiologists’ (positive hepatitis B surface antigen, hepatitis B e-antigen, and hepatitis B nucleic acid test; enzyme-linked immunosorbent assay antibody test with a high signal-to-cutoff value; recombinant immunoblot assay; and RNA test for hepatitis C).

Prevalence of self-reported current smoking (proportion of people who reported smoking cigarettes daily or on some days as of the interview day), injecting drug use (% of people who reported having used a needle to inject non-prescription drugs at least once), obesity (body mass index ≥30 kg/m^2^), diabetes (% of people who reported ever being told by a health-care professional that they have diabetes), and physical activity (% of adults who reported in the past 30 days: (1) exercising (running, calisthenics, golf, gardening, or walking, other than at their regular job) and (2) walking/bicycling >10 blocks for transportation) were obtained *via* the CHS (13). Diabetes control was measured by data from the NYC A1C Registry. We report the % of diabetic adults (history of ≥2 glycosylated hemoglobin, or A1C, test values ≥ 6.5%) who received medical care in 2012, with their last A1C measurement ≥9% (15).

As a proxy for alcohol use, clinical data on the number of patients who were hospitalized or visited an emergency department (ED) during 2014 were abstracted from the mandatory NYS hospital discharge abstract database (16), using the ICD-9 codes 291.0–291.5, 291.8, 291.9, 303.00–303.93, 305.00–305.03, 357.5, 425.5, 535.3, 571.1–4, 571.5, 571.9, 572.3, 577.1 (diagnoses of alcohol-related morbidity) (17), and of alcohol poisoning (790.3, 980, E860) (18). Only one hospitalization/ED visit per patient was counted. Approval to collect data under exempt status was obtained from Mount Sinai’s Institutional Review Board.

#### Preventive Services

Data on the availability of preventive services providing hepatitis B or C testing and treatment, hepatitis B vaccination, and syringe exchange facilities were collected from the NYC Health Map website (19), which lists names and addresses of clinics by service type. The number of services in each UHF neighborhood was obtained by matching address zip codes. The % of NYC residents who reported ever having received at least 1 dose of the hepatitis B vaccine and ever getting tested for hepatitis C was obtained from the CHS.

### Statistical Analyses

Descriptive data are presented for the entire city, and each neighborhood in the form of tables and density maps prepared using ArcGIS Desktop (version 10.3.1; ESRI, Redlands, CA). Predictors were weighted risk scores calculated for three domains of modifiable liver cancer risk factors: (1) metabolic (obesity, diabetes, and proportion of A1C ≥ 9%); (2) alcohol-related morbidity (hospitalizations, ED visits); and (3) infections (rates of newly reported hepatitis B and C cases, hepatitis B vaccination coverage, and self-reported injecting drug use). Each continuous item was given an ordinal score based on tertiles, quartiles, or a specific cutoff. For each item, neighborhoods received a prevalence score from 1 to 3 based on increasing tertiles (quartiles 1–4 for hepatitis B). Hepatitis B vaccine coverage was reverse scored to reflect a protective effect. Due to the distribution, a cutoff of <1% and ≥1% was used to score injecting drug use prevalence category as 1 or 2. Each item was also assigned a correlation score from 1 to 3 based on the strength of its correlation with liver cancer incidence (Pearson’s *r* ≤ 0.3, 0.3 < *r* < 0.5, *r* ≥ 5). Prevalence scores were multiplied by the correlation scores to obtain item scores, which were summed up to produce a risk score for each domain.

Spatial autocorrelation of liver cancer incidence was assessed using Moran’s global index (Moran’s *I* statistic) (20). A sensitivity analysis was conducted to evaluate the effect of spatial dependence by comparing linear regression models with and without a spatial lag term. The spatial lag model was run by adding a spatial weights matrix as an independent variable with weights based on inverse distances between neighborhood centroid coordinates. All spatial analyses were conducted using the spatial software GeoDa version 1.12.1.129.

The relationship between liver cancer incidence and each predictor was assessed in unadjusted and adjusted generalized linear regression models, with neighborhood as the unit of analysis (*n* = 34) (SAS Proprietary Software 9.4, TS1M1). All models met the assumptions for the specified Poisson distribution (21). Stratified analyses by prevalence of neighborhood poverty were conducted. Point estimates, 95% Wald confidence intervals, and *p*-values for the regression coefficient β were evaluated at a statistical significance level of α = 0.05 (two-sided hypothesis test).

## Results

New York City’s racial and ethnic composition includes 33% non-Hispanic White, 29% Hispanic, 23% African-American, 13% Asian or Pacific Islander, and 3% other races. Half of NYC residents (53%) are female, 21% live in poverty, and 13% are uninsured. There was considerable variation in the distribution of demographic characteristics according to neighborhood (Table 1).

**Table 1:** Distribution of sociodemographic characteristics according to neighborhood.

Neighborhood	% Male^a^	Race/ethnicity (% of population)^a^					% Living in poverty^b^	% Uninsured^c^
		White	Black	Hispanic	Asian/Pacific Islander	Other		
Kingsbridge/Riverdale	45.0	42.5	11.1	39.8	4.7	1.9	16.1	2.7
The Northeast Bronx	44.7	11.1	58.8	24.4	2.8	2.9	15.4	10.3
Fordham/Bronx Park	47.4	8.7	24.8	59.6	5.0	2.0	32.9	18
Pelham/Throgs Neck	47.0	20.4	20.7	49.7	6.6	2.7	23.2	11.8
South Bronx^d^	46.9	1.5	29.5	66.5	1.0	1.4	41.2	11.9
Greenpoint	49.5	68.1	2.9	23.0	4.1	1.9	26.5	8.6
Downtown Brooklyn/Heights/Slope	47.1	56.6	15.5	18.1	6.5	3.3	16.4	10.3
Bedford–Stuyvesant/Crown Heights	44.8	11.2	71.4	13.1	1.9	2.5	27.2	11.4
East New York/New Lots	46.1	1.9	51.2	38.8	4.7	3.4	33.4	3.8
Sunset Park	51.4	15.8	2.3	44.6	35.7	1.6	31.1	27.4
Borough Park	49.4	61.0	4.3	12.8	20.0	1.8	26.9	16.0
Flatbush	45.0	11.9	72.4	10.9	2.3	2.4	18.6	14.3
Canarsie and Flatlands	44.4	24.1	61.4	8.9	3.5	2.1	13.3	8.5
Bay Ridge/Bensonhurst	48.5	60.3	1.1	13.4	23.4	1.8	16.0	13.2
Coney Island	47.2	64.7	6.6	11.7	15.4	1.6	20.4	11.3
Williamsburg/Bushwick	48.5	14.6	30.4	48.7	4.5	1.8	31.5	10.4
Washington Heights/Inwood	48.0	15.9	12.0	68.0	2.5	1.7	25.4	18.7
Central Harlem	45.6	13.9	54.6	24.2	4.3	2.9	29.8	5.3
East Harlem	47.1	11.7	29.0	51.7	5.6	2.0	32.9	14.7
Upper West Side	45.8	67.2	7.5	14.9	7.9	2.5	11.6	7.8
Upper East Side–Gramercy^d^	45.0	75.5	3.4	7.4	11.5	2.2	8.4	7.8
Chelsea Village^d^	50.4	66.0	4.0	10.6	16.6	2.8	11.0	12.2
Union Sq–Lower Manhattan^d^	48.1	45.7	6.8	10.0	50.4	8.0	16.1	5.2
Long Island City/Astoria	49.4	46.9	6.1	27.1	16.6	3.3	16.7	9.6
West Queens	51.9	16.1	5.9	51.4	24.5	2.1	19.2	29.0
Flushing/Clearview	47.7	31.3	2.1	16.2	48.4	1.9	15.2	12.8
Bayside-Fresh Meadows^d^	47.5	44.0	5.2	12.2	36.2	2.3	11.6	7.5
Ridgewood/Forest Hills	47.7	54.5	2.0	26.1	15.5	2.0	13.1	17.6
Southwest Queens	49.0	22.6	12.4	32.7	20.2	12.1	14.3	9.5
Jamaica	46.7	7.1	53.9	18.0	14.5	6.5	16.0	19.6
Southeast Queens	46.4	13.6	54.9	11.8	14.7	5.1	7.6	9.5
The Rockaways	47.0	35.2	38.8	21.0	2.3	2.7	20.2	8.4
Northern Staten Island^d^	48.5	40.2	21.0	28.9	7.5	2.4	20.4	10.1
Southern Staten Island^d^	48.4	76.0	2.5	11.1	8.8	1.5	7.7	4.1
NYC	47.5	33.3	22.8	28.6	12.6	2.7	20.6	12.6

*^a^United States Census, 2010*.

*^b^American Community Survey, percentage with annual income below 100% of federal poverty threshold, 2010–2014*.

*^c^New York City (NYC) Department of Health and Mental Hygiene. Epiquery: NYC Interactive Health Data System—[Community Health Survey 2015] [08/28/2017]. http://nyc.gov/health/epiquery*.

*^d^Gender, race, and poverty data for combined neighborhoods are averages of constituent UHF 42 neighborhoods*.

*All percentages are age-adjusted using the 2010 US Census standard population*.

### Liver Cancer Statistics

**Figure 1:**
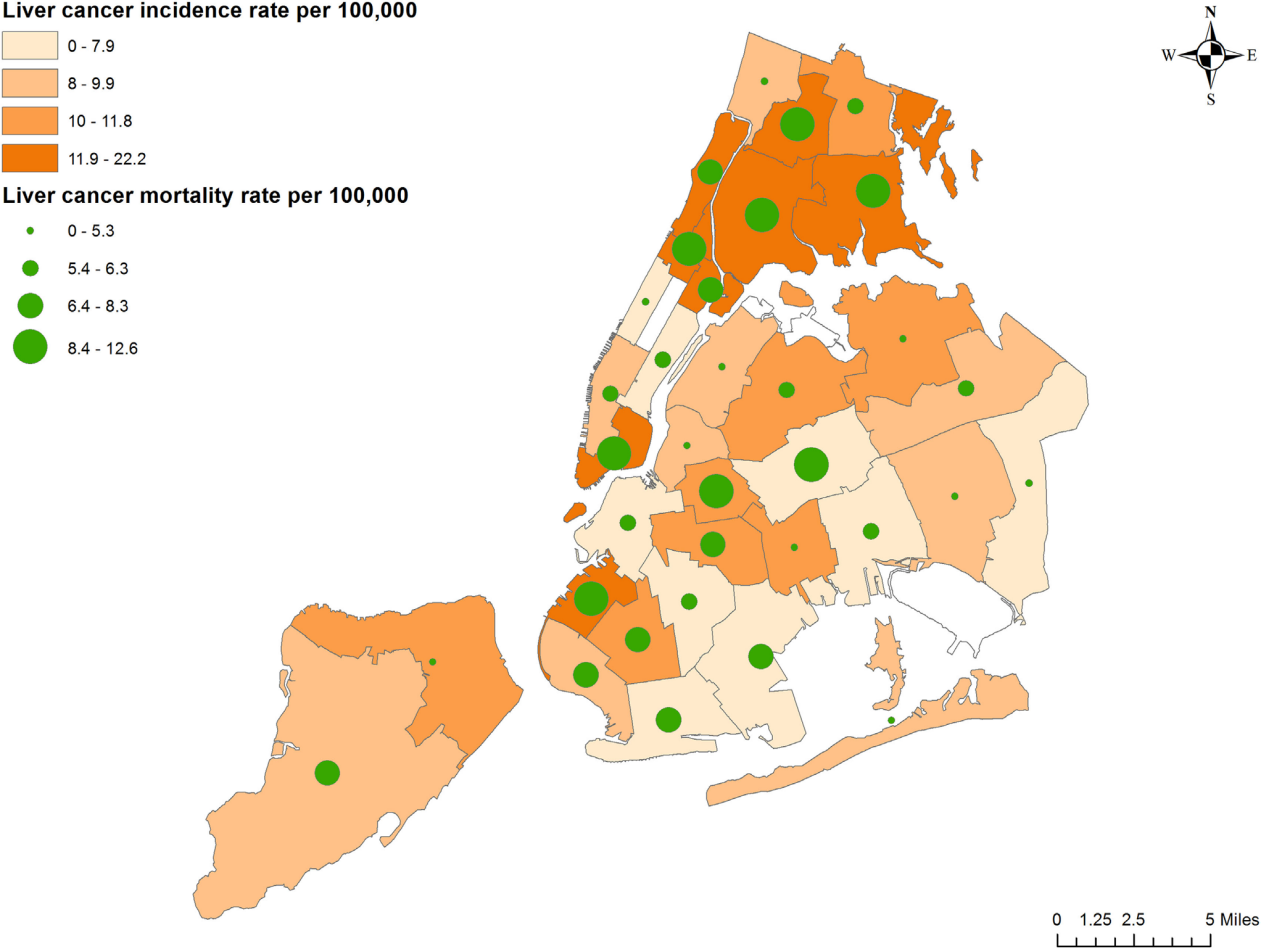
Liver cancer incidence and mortality rates according to neighborhood.

During 2007–2011, there was an average of 921.4 cases of liver and intrahepatic bile duct cancer in NYC annually. The age-adjusted incidence rates were highest in the Bronx [South Bronx (22.3), Fordham/Bronx Park (15), and Pelham/Throgs Neck (13.7)]; Manhattan [Union Square and Lower Manhattan (15.9), Central Harlem (15.8), East Harlem (15.7), and Washington Heights/Inwood (13.3)], and Brooklyn [Sunset Park (16.7)] (Figure 1). Mortality rates follow similar geographic distribution, with the highest mortality rates in Sunset Park (12.6), Fordham/Bronx Park (12.1), South Bronx (11.6), Union Square and Lower Manhattan (11.5), Pelham/Throgs Neck (11), and Central Harlem (9.2). Two neighborhoods showed high mortality rates despite relatively lower incidence: Williamsburg/Bushwick in Brooklyn (10.6) and Ridgewood/Forest Hills in Queens (9.1).

### Liver Cancer Risk Factors

The distribution of individual liver cancer risk factors is presented in Table 2. Obesity was less prevalent in NYC (24%) compared with the US average (≈38%) (4) but varied widely from 8% in the Upper West Side to 37% in East New York. East Harlem had the highest prevalence of self-reported diabetes (23%) and poor glycemic control (21%). A high proportion of poorly controlled diabetes was also observed in East New York, Bedford–Stuyvesant/Crown Heights, Williamsburg/Bushwick, the South Bronx, and Fordham/Bronx Park. There was relatively less variation in self-reported physical activity. East Harlem had the highest prevalence of self-reported injecting drug use at 4.7%, followed by Upper West Side (2.1%), and the South Bronx (1.8%). Cigarette smoking was most prevalent in Greenpoint (21%), Long Island City/Astoria, and Ridgewood/Forest Hills (19%). Finally, the mean and range of composite scores for the three modifiable risk factor domains (metabolic, alcohol, and infection) are presented in Table 3. Alcohol risk scores were moderately correlated with metabolic and infection risk scores; however, results of statistical tolerance tests did not indicate a significant threat of multicollinearity on the model estimates (22).

**Table 2:** Distribution of behavioral liver cancer risk factors according to neighborhood.

Neighborhood	Prevalence of risk factor (% of population)^h^									In the highest quartile^g^
	Current smoking^a^	IDU^b^	Exercise^a^	Walked/biked^a^	Obesity^a^	Diabetes^a^	A1C ≥ 9%^c^^,^^d^	≥1 HBV vaccine dose^e^	Ever HCV tested^f^	
Kingsbridge/Riverdale	8.3	0	83.4	73.9	33.3	8.7	15.1	51.7	28.3	
The Northeast Bronx	12.3	0	72.4	78.7	28.2	10.1	17.5	58.0	54.1	
Fordham/Bronx Park	10.4	1.39	71.2	80.0	28.6	18.4	20.2	47.8	46.3	LC, M, I, P
Pelham/Throgs Neck	16.5	0.79	73.2	76.3	29.9	11.9	19.2	51.1	42.8	LC
South Bronx^e^	17.0	1.76	70.4	82.1	34.4	20.2	20.7	47.1	52.4	LC, M, A, I, P
Greenpoint	20.7	0	77.9	85.1	26.2	9.4	16.2	37.8	41.0	
Downtown Brooklyn/Heights/Slope	13.5	0.64	81.2	92.0	16.1	4.6	17.1	60.7	36.1	
Bedford–Stuyvesant/Crown Heights	17.9	0.23	72.8	78.1	36.3	13.6	21.1	53.7	57.2	A, P
East New York/New Lots	12.3	0.19	72.3	73.5	37.1	21.7	21.5	50.1	46.5	M, A, P
Sunset Park	15.3	1.51	67.1	93.1	23.6	12.1	15.6	32.9	33.4	LC, I, P
Borough Park	15.1	0.48	69.8	77.8	16.3	8.8	13.4	45.3	26.7	I
Flatbush	9.4	1.03	72.9	82.0	35.6	13.7	19.6	51.1	47.3	
Canarsie and Flatlands	8.0	0	76.4	75.1	29.1	13.9	18.2	45.0	43.8	
Bay Ridge/Bensonhurst	15.2	0.85	73.0	83.5	21.2	10.3	12.4	33.5	30.9	I
Coney Island	18.4	0.93	68.4	82.6	26.8	13.7	12.5	39.9	36.2	I
Williamsburg/Bushwick	18.0	1.52	69.7	79.9	25.9	15.0	21.2	42.6	46.7	M, A, I, P
Washington Heights/Inwood	12.0	0.44	76.8	81.2	25.8	14.1	18.6	38.0	41.9	LC, M
Central Harlem	12.8	0.21	74.2	81.1	31.4	13.7	19.6	40.7	45.9	LC, I, P
East Harlem	16.8	4.66	65.9	83.6	27	23.1	20.7	57.3	58.3	LC, M, A, I, P
Upper West Side	13.0	2.07	91.1	90.8	7.8	6.9	14.2	63.4	37.4	
Upper East Side-Gramercy^e^	10.2	0.4	87.6	92.1	12.5	4.1	11.3	47.6	37.4	
Chelsea Village^e^	13.2	0.6	84.2	91.3	9.3	4.8	13.1	60.6	52.1	I
Union Sq–Lower Manhattan^e^	17.0	0.19	79.8	88.4	7.9	9.3	13.3	49.9	43.9	LC, A, I
Long Island City/Astoria	19.1	0.51	80.2	85.5	25.1	9.8	15.4	42.7	29.4	
West Queens	17.8	0.27	73.6	89.4	21.1	9.6	16.0	35.5	30.3	I
Flushing/Clearview	11.5	0.25	67.7	79.2	17.2	10.7	11.3	45.1	43.3	
Bayside-Fresh Meadows^e^	9.1	0.17	75.1	78.4	19.1	14.2	10.4	53.3	30.9	
Ridgewood/Forest Hills	18.8	0	70.5	83.8	17.5	5.1	13.0	40.9	36.7	
Southwest Queens	12.8	1.38	67.3	72.6	28.6	20.4	17.6	42.8	37.6	
Jamaica	6.7	0.05	67.1	79.8	30.8	13.6	17.3	40.2	44.5	
Southeast Queens	11.0	0	74.2	69.1	26.0	12.2	16.7	54.0	45.5	
The Rockaways	16.0	0.85	69.5	77.3	34.1	13.9	18.4	41.0	53.4	M, A
Northern Staten Island^e^	17.5	0	78.5	74.2	23.7	9.0	17.9	45.2	41.1	
Southern Staten Island^e^	17.5	0.37	79.3	65.5	24.6	6.2	12.4	47.9	33.1	
NYC	14.3	0.66	**74.5**	81.6	24.1	11.6	17.0	46.6	41.4	

*LC, liver and bile duct cancer incidence rate; M, metabolic risk factor score; A, alcohol risk factor score; I, infection risk factor score; P, poverty*.

*^a^New York City (NYC) Department of Health and Mental Hygiene. Epiquery: NYC Interactive Health Data System—[Community Health Survey (CHS) 2015] [08/29/2017]. http://nyc.gov/health/epiquery*.

*^b^Injecting drug use: NYC Department of Health and Mental Hygiene. CHS [2012]; public use dataset accessed on 09/08/2017*.

*^c^NYC A1C Registry, 2012; rates based on registrants reported with likely diabetes (based on a history of ≥2 A1C test values ≥ 6.5%)*.

*^d^NYC residents ages ≥18 years; rates are per 100,000 adults and are age-adjusted to 2000 Census (July 2013 NYSDH population estimates)*.

*^e^A1C registry data for combined neighborhoods are averages of constituent UHF 42 neighborhoods*.

*^f^NYC Department of Health and Mental Hygiene. Epiquery: NYC Interactive Health Data System—[CHS 2012] [09/08/2017]. http://nyc.gov/health/epiquery*.

*^g^NYC Department of Health and Mental Hygiene. Epiquery: NYC Interactive Health Data System—[CHS 2013] [09/08/2017]. http://nyc.gov/health/epiquery*.

*^h^All percentages are age-adjusted using the 2010 US Census standard population*.

**Table 3:** Association between liver cancer incidence and risk factor scores.

Risk factor domain	Scores, mean (range)	Unadjusted model				Adjusted model^a^			
		β	95% CI	% Change^b^	*p*	β	95% CI	% Change^b^	*p*
**Overall population**									
Metabolic score	9.8 (5–15)	0.0292	−0.0002 to 0.0587	3.0	0.052	0.0001	−0.0345 to 0.0348	0.0	0.995
Alcohol score	9.9 (5–15)	0.0465	0.0195 to 0.0735	4.8	0.001	0.025	−0.0112 to 0.0613	2.5	0.176
Infection score	15.3 (8–24)	0.0513	0.0256 to 0.077	5.3	<0.0001	0.0389	0.0088 to 0.069	4.0	0.011

**By poverty level** **High poverty**									
Metabolic score	12.4 (5–15)	0.0211	−0.0371 to 0.0792	2.1	0.478	−0.0862	−0.2697 to 0.0973	−8.3	0.357
Alcohol score	13.1 (5–15)	0.0186	−0.041 to 0.0782	1.9	0.541	0.0816	−0.0949 to 0.2582	8.5	0.365
Infection score	19.2 (16–28)	0.095	0.0109 to 0.179	10.0	0.027	0.1207	0.0147 to 0.2267	12.8	0.026

**Medium poverty**
Metabolic score	9.5 (5–15)	−0.0131	−0.0675 to 0.0413	−1.3	0.637	−0.0235	−0.079 to 0.032	−2.3	0.407
Alcohol score	10.0 (5–15)	0.0272	−0.0224 to 0.0768	2.8	0.283	0.0332	−0.019 to 0.0854	3.4	0.213
Infection score	14.9 (8–22)	0.008	−0.0332 to 0.0492	0.8	0.705	0.0018	−0.0401 to 0.0437	0.2	0.932

**Low poverty**
Metabolic score	8.0 (5–13)	−0.015	−0.084 to 0.0539	−1.5	0.700	−0.0075	−0.0785 to 0.0635	−0.7	0.836
Alcohol score	7.0 (5–12)	0.0106	−0.0611 to 0.0823	1.1	0.773	−0.0181	−0.1002 to 0.064	−1.8	0.666
Infection score	12.6 (8–18)	0.0504	−0.022 to 0.1228	5.2	0.172	0.0578	−0.0263 to 0.1419	6.0	0.178

*^a^All adjusted models include the following variables: metabolic score, alcohol score, and infection score*.

*^b^Percentage change in incidence of liver cancer per unit increase in risk score: calculated as (e^β^ − 1) × 100*.

### Association Between Distribution of Liver Cancer Incidence and Risk Factor Scores

Neighborhood-level data on poverty and Hispanic ethnicity were associated with high liver cancer incidence (β = 0.0277, *p* < 0.0001, and β = 0.0113, *p* < 0.0001), even after adjustment for White race and Hispanic ethnicity (β = 0.0217, *p* = 0.013). A higher proportion of foreign-born residents was correlated with higher rates of hepatitis B (*r* = 0.48, *p* = 0.0037).

Among the three modifiable risk factor domains, infection was the strongest predictor of liver cancer incidence, with an expected increase of 5.3% in incidence when the infection risk score increased by 1 (*p* < 0.0001), followed by alcohol-related morbidity (4.8% increase, *p* = 0.001) (Table 3). Metabolic risk score was also weakly but positively associated with liver cancer incidence (3% increase, *p* = 0.052). We conducted formal testing by including interaction terms between poverty tertiles and each of the three risk scores (metabolic, alcohol, and infection), and observed lack of statistical interaction. When stratified by tertiles of poverty prevalence, infection score was most strongly associated with liver cancer incidence at the high poverty level (10% increase, *p* = 0.027). Similarly, infection risk score was most strongly associated with liver cancer incidence (4% increase, *p* = 0.011), especially at the high poverty level (12.8% increase, *p* = 0.026), in models that adjusted for metabolic and alcohol risk score.

### Spatial Autocorrelation Sensitivity Analysis

The Moran’s *I* test indicated the presence of a significant positive spatial autocorrelation for the outcome, liver cancer incidence (*I* = 0.28, *p* = 0.005). Comparison of ordinary least squares regression and a spatial lag model found no meaningful effect of spatial autocorrelation on model estimates (Table S1 in Supplementary Material).

### Preventive Services

**Figure 2:**
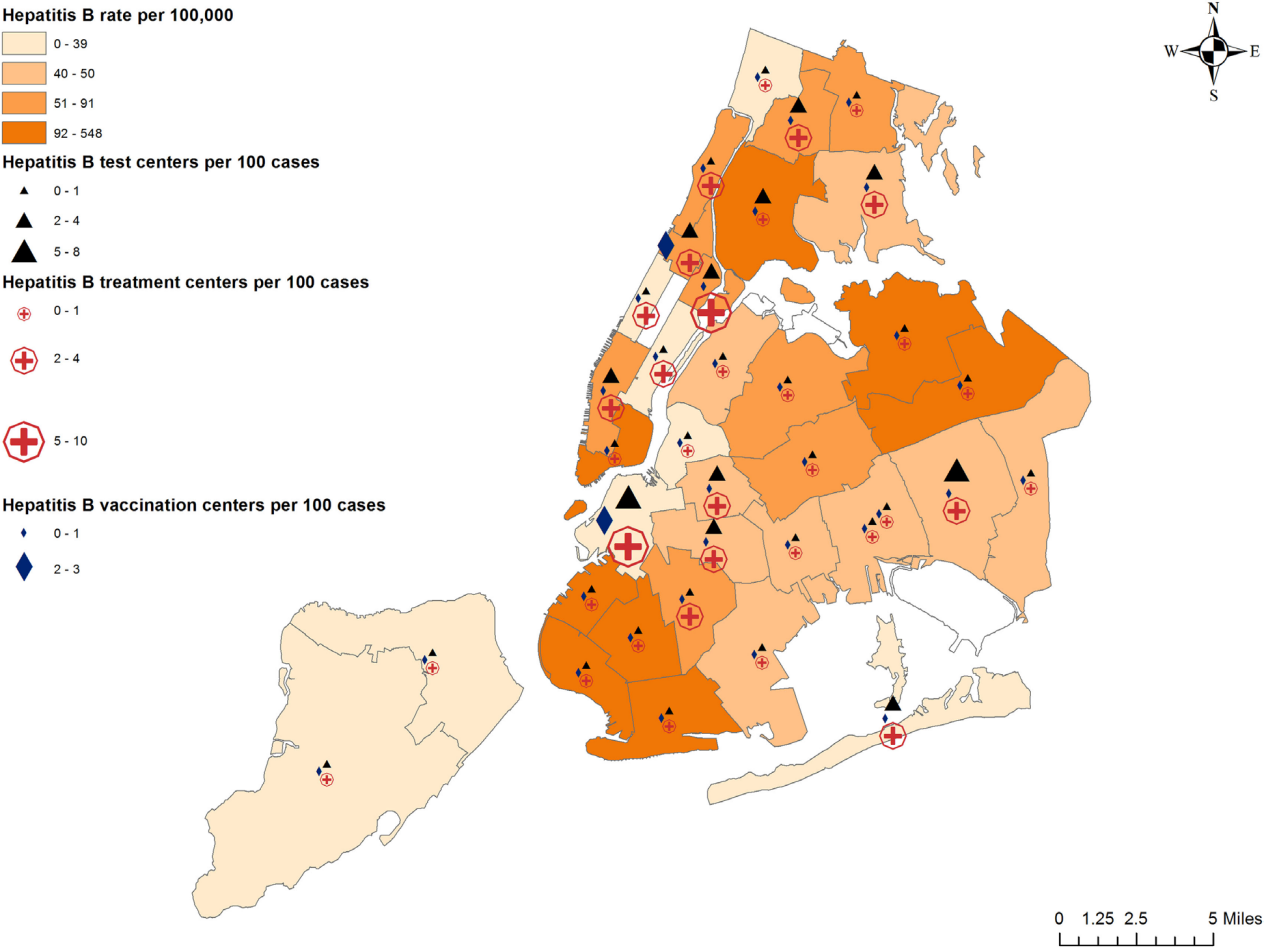
Prevalence of hepatitis B and availability of preventive services.

**Figure 3:**
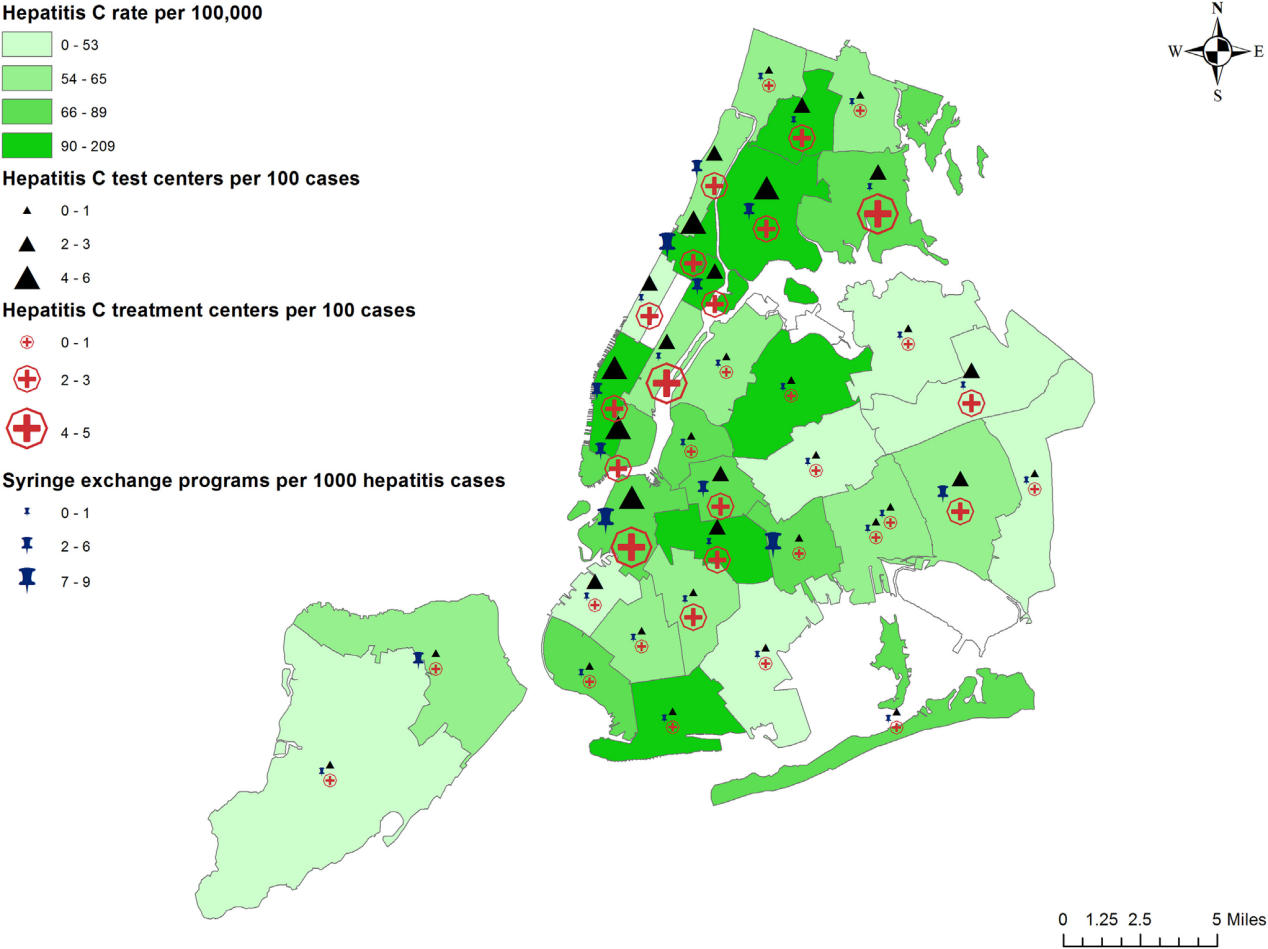
Prevalence of hepatitis C and availability of preventive services.

The number of centers offering preventive services are 80, 89, and 28, respectively, for hepatitis B testing, treatment, and vaccination; 127 and 128, respectively, for hepatitis C testing and treatment; 23 syringe exchange programs (SEPs), with multiple additional distribution locations, and hundreds of Expanded Syringe Access Program locations throughout NYC. Their availability in relation to hepatitis burden is depicted in Figures 2 and 3.

## Discussion

To the best of our knowledge, this is the first study to describe liver cancer incidence and the distribution of its underlying risk factors at a neighborhood level in NYC. In addition, this is the first study to assess the availability of hepatitis prevention and treatment services in the context of disease burden.

Results indicate that not only does NYC have higher rates of liver cancer incidence and mortality compared with NY State and the rest of the US but also large disparities exist among city neighborhoods, with incidence rates in some neighborhoods as high as those in China and West Africa (7). The most striking finding was the strong relationship between poverty, liver cancer, and its risk factors, even after adjusting for other demographics and risk factor scores. Of the three modifiable risk factor domains, infection was most strongly and consistently associated with liver cancer incidence. Rates of newly reported chronic hepatitis B and C in NYC show a gradual rise since 2013 (9, 10). This could be partially attributed to improved surveillance and test sensitivity and updated US Department of Health guidelines for Hepatitis C testing in “baby boomers” (23). However, recent changes in drug use patterns could explain the rise in both hepatitis B and C rates. The National Institute of Drug Abuse reported that the % of drug reports identified as heroin, a common injectable drug, increased from 10.4% in 2012 to 11.6% in 2013 in NYC, along with a decrease in the average age at admission to substance abuse treatment (24).

Recent immigration patterns may also contribute toward the observed increase in hepatitis B. Between 2000 and 2011, NYC has seen a 4% increase in foreign-born residents (25). Neighborhoods with the highest gains (≥5,000 people) include East and Central Harlem, Lower Manhattan, parts of the South Bronx, Sunset Park, etc. (25). The same neighborhoods have seen high immigration from three countries with high prevalence of hepatitis B (China: 5.49%, Dominican Republic: 4.09%, and Jamaica: 3.76%) (26), mirroring their own high hepatitis B rates. Chinese-born immigrants in NYC were found to have high seroprevalence of hepatitis B and increased risk for liver cancer (27). Another study found hepatitis B prevalence of 9.6% among a sample of African-born participants residing mostly in Central Harlem and the South Bronx (28). Immigrant health is an important public health issue in a diverse city like NYC. Most liver cancer risk factors are preventable, but due to poverty or other issues, health policies may not have the desired effect. Ongoing surveillance for hepatitis and effective and timely culturally and linguistically competent prevention and treatment may be the key to preventing progression to liver cirrhosis and liver cancer in NYC residents. The population of certain areas in Harlem and Bronx is ideal for exploring preventive public health strategies, and implementing surveillance programs.

Preventive and treatment services for hepatitis are available throughout the city, but not all neighborhoods with high hepatitis rates have a proportionate number of required services. We observed that high hepatitis B rates were correlated with lower vaccine coverage and lower proportion of free vaccination centers. Lower insurance coverage was also strongly correlated with lower vaccine coverage. Hepatitis B vaccination can cost $120–$370 without insurance, plus consultation/professional administration fees. This is largely unaffordable for less affluent, uninsured people. Non-monetary factors such as having a vaccinated acquaintance, perceived risk of disease, perceived vaccine safety, and provider recommendation may also influence patients’ choice to receive the hepatitis B vaccine (29). Therefore, a multi-pronged intervention is required to increase hepatitis B vaccine coverage in NYC, addressing disease-specific knowledge, access, affordability, and psychosocial factors.

While hepatitis C-related services were found to be more numerous, some neighborhoods appear to have fewer than 1 SEP per 1,000 hepatitis cases (Fordham–Bronx Park and Bedford–Stuyvesant–Crown Heights), while others (Coney Island and West Queens) have fewer than one hepatitis C testing and treatment centers per 100 hepatitis C cases. Although residents in poorer neighborhoods were more likely to get tested for hepatitis C, there is no information on how many of those who tested positive cleared the virus or received treatment. Without insurance, hepatitis C drugs for a 12-week course can cost between $39,600 and $94,500 (30, 31). Even with insurance, arranging for prior authorization of hepatitis C treatment is often time consuming and a barrier to patients starting treatment, e.g., most NYS insurance providers require a prescription to be written by or in consultation with a specialist (32). Hepatitis C treatment for those who cannot obtain health insurance is provided by the NYS Hepatitis C Patient Assistance Program HepCAP (33). However, many of them are not eligible for HepCAP, highlighting important gaps in current hepatitis C management.

This study has some limitations: as an ecological study based on the most recently available data, the neighborhood-level associations may not reflect individual risk of liver cancer; thus the results should be interpreted in a geographical context only. Surveillance data for hepatitis B and C may include people that no longer have active infection, and therefore these should not be considered incidence or prevalence rates, but simply the number of newly reported cases. Hepatitis may also be underdiagnosed due to the passive nature of surveillance data, since active testing is more costly and resource intense. Study power to detect significant associations could be restricted by small sample size (*n* = 34). Age, sex, and racial/ethnic diversity are other potential source of variation; however, due to the non-individual nature of the data and multiple race indicators, it was not possible to adjust for these variables. Alcohol risk scores were moderately correlated with metabolic and infection risk scores; however, results of statistical tolerance tests did not indicate a significant threat of multicollinearity on the model estimates. The NYC Health Map website does not provide data on private medical offices which may provide vaccinations and/or treatment whose information is not publicly available. Finally, we did not have data on homeless or incarcerated populations, who have an even higher risk of hepatitis C (34). However, this is the first study that attempts to quantify the relative role of infection, metabolic factors, and alcohol in HCC risk in a diverse environment such as NYC and highlights current gaps in hepatitis prevention services like syringe exchange and vaccination, that can be addressed by the expansion of existing services. The role of alcohol and metabolic risk factors on liver cancer in NYC warrants further study.

## Ethics Statement

This study was carried out in accordance with the recommendations of the Program for the Protection of Human Subjects Office (PPHS), Icahn School of Medicine at Mount Sinai. The protocol was approved (under exempt status) by the Institutional Review Board at the Icahn School of Medicine at Mount Sinai. A consent waiver was obtained since the data were de-identified, publicly available data.

## Author Contributions

GK, ET, and NB had full access to all the data in the study and take responsibility for the integrity of the data and the accuracy of the data analysis. Study concept and design; study supervision: ET and NB. Acquisition, analysis, or interpretation of data: GK, NE, and SE. Drafting of the manuscript: GK, ET, NB, PP, JW, and MS. Critical revision of the manuscript for important intellectual content: GK, ET, NB, NE, PP, JW, MS, JL, and SE. Statistical analysis: GK.

## Conflict of Interest Statement

JW has received research grant support and served as a consultant for Gilead Sciences, Inc. JL is a consultant for Bayer, BMS, Eisai, Celsion, Eli Lilly and has active funding for research from Bayer, BMS, and Eisai. NB has active funding from Pfizer for research. All the other authors report no conflicts of interest.
