# Supplementary material for: Liver Cancer Disparities in New York City: A Neighborhood View of Risk and Harm Reduction Factors
Source: Front Oncol. 2018 Jun 14;8:220. doi: 10.3389/fonc.2018.00220 (PMC6011126; doi:10.3389/fonc.2018.00220)
Supplement: Supplementary file 1 [file table_1.docx]

Supplementary Table 1. Comparison of linear regression models with and without a spatial lag term

| **Risk factor domain** | **Ordinary Least Squares model** | | | | **Spatial lag model^1^** | | | |
| --- | --- | --- | --- | --- | --- | --- | --- | --- |
|  | **Unadjusted model** | | **Adjusted model^2^** | | **Unadjusted model** | | **Adjusted model^2^** | |
|  | **β (SE)** | **p** | **β (SE)** | **p** | **β (SE)** | **p** | **β (SE)** | **p** |
| Metabolic score | 0.452 (0.16) | 0.001 | 0.036 (0.16) | 0.818 | 0.438 (0.14) | 0.002 | 0.038 (0.13) | 0.770 |
| Alcohol score | 0.579 (0.13) | <.001 | 0.253 (0.16) | 0.132 | 0.562 (0.11) | <.001 | 0.268 (0.14) | 0.048 |
| Infection score | 0.583 (0.10) | <.001 | 0.433 (0.13) | 0.002 | 0.549 (0.09) | <.001 | 0.387 (0.11) | <.001 |
| ^1^Weighted using inverse distances between neighborhood centroid coordinates  ^2^All adjusted models include the following variables: metabolic score, alcohol score, infection score | | | | | | | | |
